# Resilience and vulnerabilities of urban food environments in the Asia‐Pacific region

**DOI:** 10.1111/mcn.13513

**Published:** 2023-04-25

**Authors:** Emily Rousham, Martyn Clark, Michelle Latham, Swan Pyae Oo, Sonja Read, Paula Griffiths, Jessica Blankenship, Sophie Goudet

**Affiliations:** ^1^ Centre for Global Health and Human Development, School of Sport, Exercise and Health Sciences, Loughborough University Loughborough UK; ^2^ Nutrition Research, Dikoda London UK; ^3^ Nutrition Section UNICEF East Asia and the Pacific Regional Office Bangkok Thailand

**Keywords:** COVID‐19, diet, low‐income and middle‐income countries (LMICs), nutrition transition, public health, urbanisation

## Abstract

Rapid urbanisation in the Asia‐Pacific region is associated with complex changes to urban food environments. The impact of changing food environments on food purchasing and consumption and the diets and nutritional status of vulnerable groups, especially women and young children, is not well researched in low‐ and middle‐income country cities. This paper aimed to examine: the risks and opportunities for healthy diets for low income populations offered by modernising urban centres; the concept of food deserts in relation to urban food environments in the Asia‐Pacific region and how these could be mitigated; and measures to strengthen the resilience of food environments in the region using a case study of the impact of COVID‐19 on informal food vendors. Our findings indicate that the dynamic changes in urban food environments in the Asia‐ Pacific region need to be understood by examining not only modern retail food outlets but also wet markets and informal food outlets, including street foods. Efforts should be made to ensure both modern and traditional outlets provide complementary platforms for convenient, affordable and accessible nutritious foods for urban populations. The resilience of urban food environments to environmental, physical and socio‐economic shocks can be strengthened by shortening food supply chains and maximising food production in cities. Support mechanisms targeting urban informal food outlets and street vendors can also strengthen resilience and improve food security. Further research is needed on the impact of urbanising food environments on consumer choices, preferences, diets and health outcomes.

## Key messages

- Urban food retail outlets in low‐ and middle‐income countries in Asia and the Pacific are undergoing rapid modernisation which brings risks as well as opportunities for healthy diets among vulnerable groups including women and children.
- With rapid modernisation of food retail outlets, steps should be taken to prevent food deserts and food swamps in low income urban neighbourhoods.
- Informal food retailers and street food vendors, a key component of urban food environments in the region, were particularly adversely affected during the COVID‐19 pandemic.
- Shorter food supply chains and the promotion of food production in cities and surrounding areas can further strengthen the resilience of urban food environments.

## Graphical abstract

This paper highlights the dynamic changes in urban food environments in the Asia Pacific. Whilst modern retail food outlets are increasing, wet markets and informal food outlets, including street foods, continue to play an important role in nutrition and food security. Steps should be taken to ensure both modern and traditional outlets provide complementary platforms for convenient, affordable and accessible nutritious foods for urban populations and particularly for women and young children.

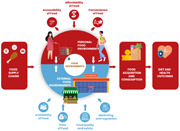

## 1 INTRODUCTION

**Figure 1:**
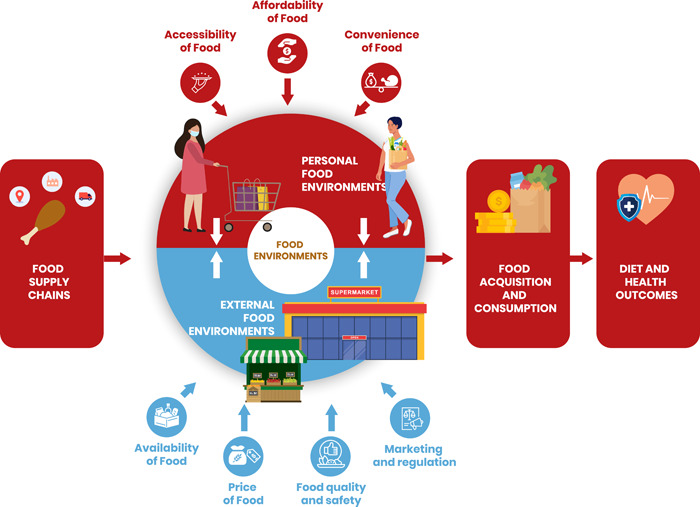
The urban food environment within a food systems framework.

The food environment refers to the physical, economic, sociocultural and policy conditions that shape food access, food affordability, food safety and food preferences (FAO IFAD UNICEF WFP & WHO, 2021). The food environment can also be viewed as the interface between the food system and the diet of the population (FAO, 2016). Within the food environment lie external and personal domains, as illustrated in Figure 1. The external domain of the food environment relates to food availability, prices, vendor characteristics (including food quality and safety) and marketing and regulation (Raza et al., 2020; Turner et al., 2018; UNICEF & GAIN, 2019). The personal domain includes accessibilty, affordability and convenience as well as desirability of foods for the consumer (Raza et al., 2020; Turner et al., 2018). Both the external and personal food environments contribute to food acquisition and consumption which in turn determine nutrition, food security and dietary and health outcomes (Hawkes et al., 2015). The bidirectional relationships between customers and the retail food environments are also important, such that customer choices and experiences can influence the food environment and vice versa (Turner et al., 2020; Winkler et al., 2020). These reciprocal interactions between the personal and external food environment are also featured in Figure 1.

Urbanisation and the rise in incomes associated with urban living are central to the food choices made by consumers, as well as the types of foods available. In high income countries (HICs), the food environment tends to be dominated by supermarkets and modern convenience retailers as the primary source of fresh, nutritious foods (Bodor et al., 2010). In many low‐ and middle‐income countries (LMICs), however, informal food outlets and non‐market‐based food sources (self‐production, food transfers) are central components of the urban food environment (Turner et al., 2018).

Countries in the Asia‐Pacific region are experiencing rapid urbanisation with approximately 43% of the region's total population residing in urban areas in 2019 (United Nations, 2019). This rapid urbanisation impacts diets through changes to food environments throughout the region. Urbanisation is associated with rapid increases in the number and density of modern food retail outlets, with varying rates of growth depending on stage of economic development (Herforth & Ahmed, 2015). Urban food environments can be characterised by increasing diversity of food choices and food retailers. However, this can also bring greater access to processed, packaged and ready‐made, energy‐dense food, in addition to increased consumption of food produced, processed, and cooked by others (Baker & Friel, 2016; Popkin, 2015).

As the number of modern food outlets increases in urban food environments, there is a risk of creating food deserts or food swamps in low income areas. The concept of food deserts originated from the Global North to describe the lack of affordable and accessible nutritious foods in low income, urban environments (Beaulac et al., 2009; Dutko et al., 2012). Food deserts are characterised as having large or sparse populations of low‐income communities with high levels of unemployment, inadequate access to transportation, and a paucity of food retailers that provide fresh and healthy produce at affordable prices (Beaulac et al., 2009; Dutko et al., 2012). The concept encapsulated the challenges of long distances to supermarkets or grocery stores and lack of transportation or poor transport infrastructure. Access to fresh foods including fruits and vegetables is often limited in food deserts (Hendrickson et al., 2006), whereas fast‐food restaurants may be abundant (Dutko et al., 2012). In US cities, food deserts tended to have a large proportion of vulnerable ethnic minority groups and strong associations with poverty (Dutko et al., 2012). A related concept of food ‘swamps’ has been used to describe areas with a high density of outlets selling foods that do not contribute to a healthy diet, such as confectiouary, junk foods, highly‐processed, or high energy foods (Crush & Si, 2021; Wagner et al., 2019), and this is often an accompanying feature of food deserts. The application of the concepts of food deserts and food swamps to the specific contexts of low income urban communities in Asia and the Pacific region warrants further consideration to identify ways to prevent these being created as LMICs undergo rapid urbanisation.

Rapid urbanisation and the associated changes in food environments have implications on the food security and nutrition of populations, especially vulnerable groups including women and young children. This brings concerns about the increasing prevalence of multiple forms of malnutrition such as stunting, micronutrient deficiencies as well as overweight or obesity (FAO UNICEF WFP & WHO, 2021; Vilar‐Compte et al., 2021; Westbury et al., 2021).

Following the recent COVID‐19 pandemic, it is important to understand the impact of large‐scale shocks on urban food environments and the associated effects on nutrition and food security. During the pandemic, urban food environments were affected by disrupted food supply chains, worsening of food affordability, and were particularly sensitive to fluctuating food prices (Bene et al., 2021; WFP & Dikoda, 2022). Further, across the region, the price gap between healthy and unhealthy foods has widened over the last two decades which is a further barrier to healthy diets among the urban poor (Farrell et al., 2021).

To assess the resilience and vulnerabilities of food environments in the Asia‐Pacific region, we focus on the opportunities and threats to healthy diets in the context of urbanisation and modernisation of food environments. This paper aimed to (1) assess the growth, availability and accessibility of supermarkets as proxy indicators of the modernisation of the food retail environment in the Asia‐Pacific region compared to other regions; (2) examine how the concept of food deserts applies to the the Asia‐Pacific region, noting how this may differ from traditional definitions; and (3) identify measures for strengthening the resilience of urban food environments to physical, environmental or socio‐economic shocks using a case study of the impact of COVID‐19 on informal food vendors in three cities.

## 2 METHODS

To investigate the three aims, we used secondary data analyses; conducted a critical review and narrative synthesis of literature, and used unpublished data from World Food Programme & Dikoda (WFP & Dikoda, 2021) for the case study.

For aim 1, we used secondary data extracted from the Food Systems Dashboard (GAIN & Johns Hopkins University, 2021) to undertake a regional analysis of the number and rate of growth of supermarkets in the Asia‐Pacific region compared to other global regions. These were used as indicators of a modernising retail environment. Rate of growth was assessed using the average percent change in number of supermarkets over a 5‐year period (2012–2017) (GAIN & Johns Hopkins University, 2021). Then, we conducted a country‐level analysis within the Asia‐Pacific region on the same indicators of number of supermarkets and 5‐year rate of growth of supermarkets. For both analyses, the definition of supermarkets was based on the original data source, as ‘Retail outlets selling groceries with a selling space of between 400 and 2500 square metres’ (GAIN & Johns Hopkins University, 2021). Data on other types of modern food retail outlets were not reported. This definition of supermarkets does not include discount retailers, and smaller convenience stores or independent grocery stores, but provides a consistent indicator across countries of one type of modern retail outlet.

Next, we extracted and analysed open access data to estimate the accessibility of supermarkets as a proxy indicator of modern food retail outlets in cities within the Asia and the Pacific region. Supermarket accessibility was assessed as the percentage of the city population living within a 20 min walk of a supermarket. We used OpenStreetMap (http://www.openstreetmap.com) to estimate the number of supermarkets in 2021 across major cities in the region. In all cases except one this was the capital city. In the case of Sri Lanka we included the former capital city, Colombo as the major centre. Supermarkets were identified using the class ‘shop type’ and sub‐class ‘supermarket’ in OpenStreetMap. This is the most reliable search term in OpenStreetMap because there is no single search term/classification that would capture other types of convenience stores or modern retailers within the class of ‘shop’. The spatial extent of each city was established by reviewing official development plans and then a spatial join of these boundaries against the OpenStreetMap database was undertaken with the results filtered to include features of only ‘shops’ with sub‐class ‘supermarkets’.

Distance to supermarkets was estimated using the number of supermarkets enumerated on OpenStreetMap in each city within an approximate radial boundary of 50 km around a centre point for each city. Population estimates were extracted from www.worldpop.org (2020) using the same 50 km buffer. In the case of the Pacific Island countries (Samoa, Micronesia, Vanuatu, Solomon Islands and Papua New Guinea) we used a 25 km buffer for the enumeration of supermarkets and total city population becauase of the smaller geographic scale of cities in these countries. These estimates were used to calculate the number of people living within a 20‐min walk of a supermarket as a proportion (%) of the total population of the city. We also calculated the number of people living within a 5 min walk of a supermarket using the same method. Enumeration of supermarkets using OpenStreetMap is likely to underestimate actual numbers and completeness of mapping is likely to vary across countries, but this was selected as a low‐cost, freely available data source for countries across the region, to test the broad approach described here.

For aim 2, we reviewed peer‐reviewed research articles, reviews and included relevant grey literature from intergovernmental organisations, international non‐government organisations and government departments to assess how the concept of food deserts applies to cities in the Asia‐Pacific region. Scoping searches were conducted using PubMed and Google scholar using key search terms (food deserts; food security; food environment; food purchasing; street food; food vendors; urban agriculture; COVID‐19; urban nutrition; urban slum; urban poverty; urban poor; Asia; Pacific and country names of members states with the FAO Asia‐Pacific region) with publication dates from 2010 onwards. Subject experts in agriculture, food systems and nutrition for the Asia‐Pacific region were consulted for sources of grey literature. We included searches of reference lists of publications to include key publications data before 2010.

For aim 3, the case study of the impact of COVID‐19 on urban food outlets and informal food vendors, we used secondary data from a rapid market assessment conducted in 2020 within informal settlements in three mega‐cities: Jakarta in Indonesia, Dhaka in Bangladesh and Quezon City in the Philippines. The assessments in each city followed on from previous programmatic work conducted by World Food Programme (WFP & Dikoda, 2021). The survey methods were informed by the Emergency Market Mapping and Analysis (EMMA) toolkit and involved consultation with stakeholders including food vendors, microfinance and financial institutions. The work was conducted for potential future urban programming by World Food Programme and partner organisations. The rapid market assessment included a survey of food outlets and informal vendors within an approximate 1 km radius area of a low income/slum area to assess: the impacts of COVID‐19; vendor needs; and barriers to finance and technical assistance. Semi‐structured interviews were conducted with microfinance and financial institutions and other actors (e.g., one local industry association, a local intervention partner) to investigate the availability of financial support for food retailers. An urban stakeholder mapping exercise was carried out including local governance and national level actors whose work influences the urban food environment as well as humanitarian organisations working in the area. A total of 238 food outlets and vendors (*n* = 30 in Jakarta, *n* = 147 in Dhaka, *n* = 61 in Quezon City) were surveyed and 23 semi‐structured interviews conducted. Further details of the surveys and data generated are available upon request from Dikoda.com.

## 3 RESULTS

### 3.1 Modernisation of retail outlets in urban food environments in the Asia‐Pacific region

**Figure 2:**
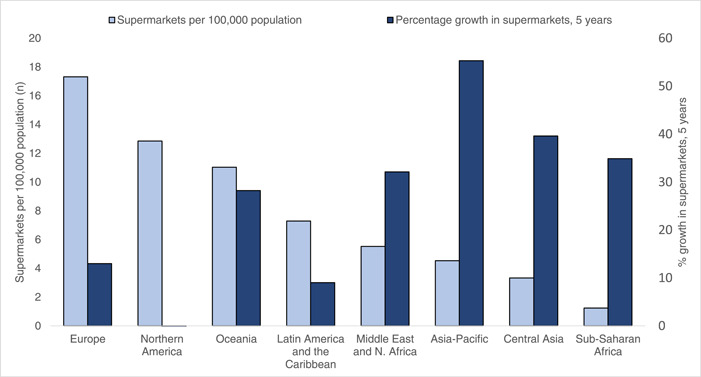
Growth of supermarkets in the Asia‐Pacific region compared to other global regions (% increase from 2012 to 2017) (y2, right axis) and the average number of supermarkets per 100,000 population (y1, left axis) for each region. Data source: (GAIN & Johns Hopkins University, 2021).

The regional analysis of supermarkets using secondary data indicates that growth of supermarkets in the Asia‐Pacific region surpassed all other global regions, with an average 55% increase in the number of supermarkets from 2012 to 2017 (Figure 2). Conversely, the Asia‐Pacific region had the third lowest number of supermarkets in proportion to the population, at 4.5 per 100,000 population, compared to 17.3 and 12.8 per 100,000 population for Europe and North America respectively. Central Asia and sub‐Saharan Africa were the regions with the lowest supermarket proportion (3.3 per 100,000 and 1.2 per 100,000, respectively) (Figure 2).

**Figure 3:**
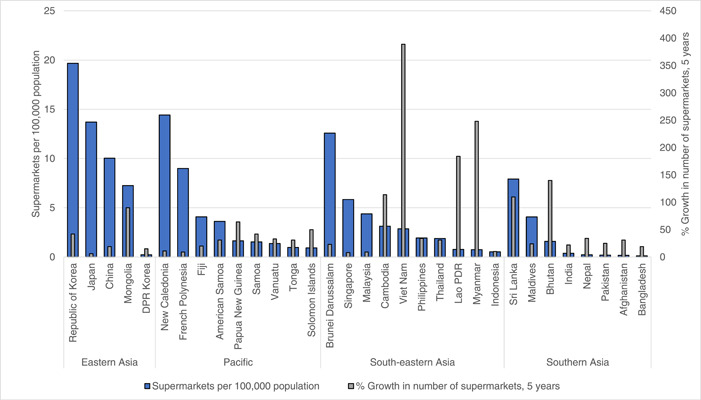
Number of supermarkets per 100,000 population (left‐hand vertical axis) and percentage increase in supermarkets 2012–2017^a^ (right‐hand vertical axis) for 33 countries in the Asia‐Pacific region. ^a^Average percent change in number of supermarkets over a 5‐year period (2012–2017) extracted from Food Systems Dashboard. Data source: (GAIN & Johns Hopkins University, 2021).

A country‐level analysis of data revealed a wide range in the availability of supermarkets across the Asia‐Pacific region (Figure 3). Supermarket availability was greatest in the HICs of Republic of Korea (19.7 per 100,1000 population), New Caledonia (14.4 per 100,000), Japan (13.7 per 100,000) and Brunei Darussalam (12.6 per 100,000), followed by China (10.0 per 100,000), an upper middle‐income country.

In contrast, the percentage growth rates in number of supermarkets were highest among several lower‐middle income countries indicating a dynamic shift towards modernisation of the food environment (Figure 3). Vietnam had the greatest expansion of supermarkets at 389% growth from 2012 to 2017, followed by Myanmar (248%), Lao PDR (184%), Bhutan (140%) and Cambodia (114%). Growth of supermarkets was lowest in countries with more highly developed economies, such as Malaysia (9%), French Polynesia (8%), Singapore (8%) and Japan (6%) indicating that expansion of modern food retailers in these countries is reaching saturation (Figure 3). The south Asian countries of Afghanistan, Bangladesh, India, Nepal all had a relatively low proportion of supermarkets per capita (range 0.11 per 100,000 in Bangladesh to 0.38 per 100,000 in India) and also a relatively low percent growth in supermarkets (ranging from 19% in Bangladesh to 38% in India) (Figure 3).

**Figure 4:**
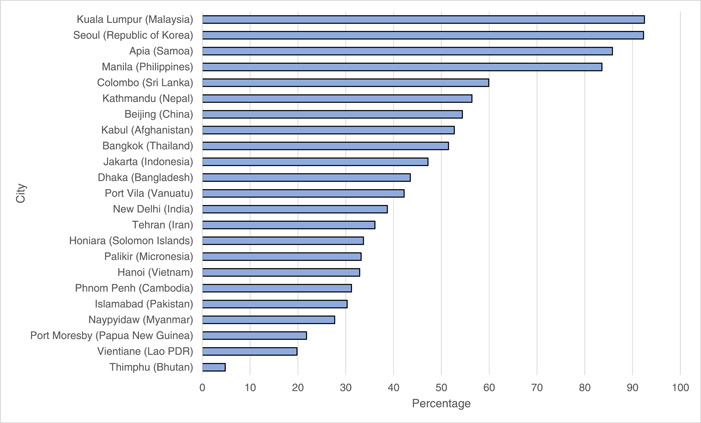
Access to supermarkets in cities in the Asia‐Pacific region estimated as the percentage of the total city population (in a 50 km boundary of the city centre) living within a 20 min walk of a supermarket. Data sources: Supermarket numbers estimated from OpenStreetMap (2021). Population figures were obtained from www.worldpop.org using 2020 estimates within a 50 km buffer of each city or 25 km buffer in the case of the Pacific Island States.

Accessibility of supermarkets at the city level in the Asia‐Pacific region was estimated using a proxy measure of the proportion of the urban population within a 20 min walking distance to supermarkets estimated using spatial mapping. Figure 4 shows the percentage of the population within a 20 min walk of a supermarket for 23 major cities (all capital cities, except Colombo in Sri Lanka, a former capital city). This figure highlights the very different profiles of supermarket access across the region. Cities with a low proportion of the population living within a 20 min walk of a supermarket were Thimphu, Bhutan (4.8%), Vientiane, Lao PDR (19.8%), and Port Moresby, Papua New Guinea (21.8%). Cities with the highest proportion of the population within 20 walking distance to a supermarket were Seoul, Republic of Korea, and Kuala Lumpur, Malaysia (both 92%). We also calculated the number of people living within a 5‐min walk of a supermarket which yielded a similar pattern of variation (see Table S1).

### 3.2 Food deserts and food swamps in LMICs in the Asia‐Pacific region

**Figure 5:**
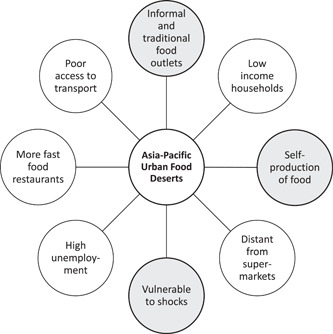
Characteristics of urban food deserts showing typical features (white circles) and additional features for consideration in the Asia‐Pacific region (shaded circles).

Based on the literature on food deserts and food swamps in HICs, we reviewed how these characteristics applied to urban food environments in the Asia‐Pacific region. We identified three features of food environments among urban low income neighbourhoods in the Asia‐Pacific region that distinguished them from traditional definitions of food deserts. These were: (i) the availability of traditional and informal food outlets, (ii) the ability to self‐produce food, and (iii) high vulnerability to environmental and socio‐economic shocks. Figure 5 summarises the typical characteristics of food deserts of the Global North (Beaulac et al., 2009; Dutko et al., 2012) with the three additional features relevant for low income urban centres in the Asia‐Pacific region. These three features are considered in turn below.

#### 3.2.1 Traditional markets and informal food outlets in the Asia‐Pacific region

The array of traditional markets and informal food retail outlets form a prominent feature of urban food environments in the Asia and the Pacific region. Traditional wet markets are typically public marketplaces selling fresh produce, meat and fish (IFAD, 2020). These are common shopping areas for urban residents to buy fresh meat and vegetables, often from local farms and producers. These markets are often the preferred destination for large segments of the urban population for their affordable, fresh and nutritious food. Wet markets tend to offer a greater diversity of fresh fruits, grains and vegetables compared to supermarkets (IFAD, 2020).

Similarly, informal food stalls and mobile food outlets are ubiquitous in large cities and small towns across the Asia‐Pacific region and make a vital contribution to food security for urban dwellers, especially the urban poor (FAO, 2007). The informal food sector covers small producers, manufacturing enterprises, traders and service providers related to food (FAO, 2007). The disaggregation of food outlets into the formal and informal market food environments is broadly based on the extent of regulation by formal governance structures. Modern retailers (supermarkets, convenience stores and grocery shops) largely fall under formal governance whereas informal markets, in general, lie outside formal governance (Ahmed et al., 2021). Informal market food environments have been defined as including wet markets, street vendors, kiosks and mobile vendors or hawker stalls (Ahmed et al., 2021).

Within the informal food retail sector, street foods play a central role across the Asia‐Pacific region. Streets food are defined as ‘ready‐to‐eat foods and beverages prepared and/or sold by vendors or hawkers especially in the streets and other similar places’ (FAO, 1989). Street food outlets create employment, including for women and those working in the informal economy, and play an important role in local culture (Roever, 2014). Street foods can also bring nutritional benefits by providing accessible, affordable foods with ready availability for time poor workers, and as such can contribute to food security for the urban poor. The nutritional and health concerns surrounding street foods include the risk of microbial contamination, poor hygiene in food preparation and handling, complex or non‐existent licensing systems, and weak regulation and food safety inspections (FAO, 2019). Street food vendors may also sell highly‐processed or unhealthy foods and beverages (WHO, 2019). While some urban planning policies have curtailed the operation of street food vendor businesses, other initiatives have provided legal protection of street food vendors; an acknowledgement of the important role that these outlets play in the urban food environment. In India and the Philippines street food vendors were legalised in 2001. In Indonesia, the state government in Jakarta waived license fees for street vendors in 2013 and has included areas for hawkers in the city's Spatial Plan 2030. Further work is needed to classify and understand street foods and the impact of the informal sector on people's food purchasing, consumption and nutritional status (Abrahale et al., 2019; WHO, 2019).

#### 3.2.2 Urban agriculture and self‐production of food across cities in the region

Urban agriculture and self production of foods is a key strategy to mitigate the risks of food deserts as well as increasing the resilience of urban food envrionments. A priority in the FAO Urban Food Agenda is to enhance local food production in cities and the surrounding regions (FAO, 2019). Local food production in cities can shorten food chains which, in turn, can create retail food environments that are more resilient to socio‐economic, political or environmental disruptions and economic shocks (FAO, 2019). Other benefits of urban agriculture and food production include reduced packaging, food losses and waste (FAO, 2019) as well as providing fresh, nutritious foods. The challenges of urban agriculture in cities across the Asia‐Pacific region, however, are the high population densities, weak planning systems and high demands on land for residential and retail purposes. The scarcity of land around urban centres means that agriculture has to be approached differently to rural agriculture, achieving high productivity per unit of land and efficient energy use. This brings benefits of providing high quality, fresh produce, whilst also supporting urban employment. This in itself can reduce poverty in urban low income areas (Akaeze & Nandwani, 2020). Local food production can also be supporteed by linking small farmers to urban supermarkets in the locality or creating hubs for small producers to link with modern retail outlets (Reardon et al., 2012). Where land is scarce, city governments could promote the use of public spaces, community centres and school premises for food production for the urban poor. Cities can promote school garden programmes which play an important role in nutrition and food security, with examples in Sri Lanka (Eigenbrod & Gruda, 2015). Innovations in vertical farming and the use of alternative spaces in the built environment provide further opportunities for self‐production. Japan has a long standing practice of urban agriculture employing roof‐tops or green walls of edible species as part of urban regeneration projects (Moreno‐Penaranda, 2011). The peri‐urban agriculture sector in Malaysia employs diverse technologies (hydroponics, rain shelters, netted structures) to support high productivity within limited spaces (Man et al., 2017).

Smart development approaches also provide opportunities to increase urban agriculture and align with urban planning and development goals, ideally benefitting disadvantaged or vulnerable groups (FAO, 2019). Food supply chains are being digitised with technologies such as cloud computing, artificial intelligence and the Internet of Things (IoT) (FAO, 2019) with many initiatives being led in Singapore. These technologies can open up opportunities for smallholders and other small‐medium food enterprises.

#### 3.2.3 Vulnerabilities of urban food environments to shocks and adverse events: The impact of COVID‐19

The COVID‐19 pandemic in 2020–21 threw a spotlight on the vulnerability of food systems and urban food environments to external shocks. In the first half of 2020, millions of households were pushed into acute food insecurity (Osendarp et al., 2021; United Nations, 2020). In the first wave of the pandemic, closure of restaurants, canteens and street food outlets and restrictions of sales in public spaces had major impacts on urban food environment in LMICs (FAO, 2020b). These closures created limited options for food purchasing and exemplify the risks of creating food deserts. The unprecedented events of the pandemic have led to heightened awareness of the need to build resilient urban food environments especially in LMICs (Bene et al., 2021; O'Meara et al., 2022).

Women and young children were particularly at risk of worsening nutritional status during the COVID‐19 pandemic (Headey et al., 2020). Reduced access to essential health and nutrition services placed further risks on women and young children (Kalbarczyk et al., 2022; Osendarp et al., 2021; Picchioni, Goulao, & Roberfroid, 2021). The pandemic is also likely to have exacerbated existing gender inequities for women and girls (Kalbarczyk et al., 2022). The early phases of the pandemic led to high unemployment and loss of income. Food became less affordable and physical access to the food environment was limited by lockdown measures and fears of contagion or infection with COVID‐19 (Bene et al., 2021; HPLE, 2020). The combination of reduced food choices, food affordability and restrictions on food acquisition led to food insecurity and worsening diet quality (Laborde et al., 2021).

Countries in the Asia‐Pacific region experienced shortages of labour in local agriculture and food‐related activities that further impacted on urban food environments (FAO, 2020b). In some countries, food supply affected urban dwellers more acutely than rural dwellers because of longer food supply chains and interrupted transport routes to urban areas (Kang et al., 2021). Surveys of eight cities across the region (Chittagong, Cox's Bazar and Dhaka, Bangladesh; Jakarta, Indonesia; Kabul, Afghanistan; Peshawar, Pakistan; Phnom Penh, Cambodia; and Quezon City, Philippines) showed that food became less affordable for low income urban residents due to the combined effects of increased food prices and loss of income (WFP & Dikoda, 2021). Food insecurity was particularly common among informal sector workers who experienced loss of income and were often not registered for social protection programmes (WFP & Dikoda, 2021). In Pacific Island states, tight restrictions on food imports and the closure of informal markets during COVID‐19 had far‐reaching impacts (Farrell et al., 2020). In the Solomon Islands, market closures disproportionately affected women, who made up the majority of market sellers (FAO, 2020a). Reduced availability and affordability of local and imported food led to local adaptations and coping stratgies such as road side sales of food, informal bartering and food exchange systems, some of which used online marketplaces (FAO, 2020a). City level governments played an important role in developing actions to mitigate these disruptions. For example, Dhaka City Corporation collaborated with the government ministries to distribute food assistance and worked with local community organisations to identify and distribute food baskets to the most vulnerable at the height of food scarcities (FAO, 2020b). A number of city governments in Asia and the Pacific directly purchased food from local producers. In Davao city in the Philippines, the city government purchased, repackaged and distributed food to the most vulnerable to assist both smaller farmers and households in urban areas (FAO, 2020b).

### 3.3 Case study of the impact of COVID‐19 on informal vendors in three mega‐cities in the Asia‐Pacific and future measures to build resilience

During the first year of the COVID‐19 pandemic, rapid market assessments were conducted in informal settlements in three mega‐cities; Jakarta in Indonesia, Dhaka in Bangladesh and Quezon City in the Philippines (WFP & Dikoda, 2021). The surveys aimed to understand the market environment and stakeholder needs and then produce actionable operational recommendations for future potential urban programming in the three cities. A specific aim was also to understand the impact of COVID‐19 on the informal food sector in terms of availability, access, utilisation of food and access to financial support.

The rapid market assessment findings revealed that in all three cities, government COVID‐19 restrictions had a major impact on the operational environment of food businesses. The reduction in demand from consumers had a crippling effect on the informal food vendor market. Several factors contributed to reduced consumer demand. First, low employment of daily workers led to loss of income which decreased consumer capacity to buy food products; second, office and workplace closures in urban centres meant that there were fewer workers buying meals and ready‐to‐eat foods during the working day (WFP & Dikoda, 2021); and finally, consumers turned to vendors closest to their place of residence to minimise time and distance spent outside and in human interactions due to perceived risks of COVID‐19 transmission.

In Dhaka and Quezon City, informal street vendors were more adversely affected than formal small‐to‐medium enterprises. Informal and street food vendors also had fewer options for adapting to the conditions during the pandemic. For example, the informal vendors in the surveyed areas generally had not capitalised on the growing online market, which other enterprises relied upon during the crisis. In Jakarta, street food vendors may have been more resilient due to a supportive legal framework. Despite overall reductions in demand for some informal food outlets, street food vendors continued to be an important source of food for poorer sectors. In the surveyed areas there were relatively few modern food retailers which made the normal functioning of informal food outlets particularly important for the urban food environment.

These market assessments highlighted that the informal sector presents an affordable option for local populations as well as providing livelihoods for individuals within the community, but these businesses were put at risk by the pandemic. Recommendations for interventions have been put forward to strengthen the food environment in the three cities with a focus on informal food vendors (Table S2). Proposed intervention objectives included: improving purchasing power of consumers for example through cash or voucher schemes for those who lost their jobs; supporting consumer confidence in informal retailers through food safety and hygiene measures to prevent transmission during the pandemic; capacity building and increasing resilience of street food vendors through training in online retail technologies; increasing market linkages for small retailers; and increasing registration of street food vendors. Some intervention objectives applied to just one of the surveyed cities, while other objectives applied to all three cities (Table S2). Many of the identified actions would benefit the informal food market sector. For vendors selling nutritious foods, this would also increase consumer access to healthy diets.

## 4 DISCUSSION

### 4.1 Growth of supermarkets and impact of modern retail outlets on the food environment

Our analysis revealed that the Asia‐Pacific region had among the lowest availability of supermarkets relative to population number, but the fastest growth rate of supermarkets compared to other global regions from 2012 to 2017. At the country level, data indicate that several lower middle income countries in the region, notably Vietnam, Myanmar, Lao PDR and Cambodia showed a rapid 5‐year growth in the number of supermarkets. This points to a dynamic urban food environment moving towards a greater presence and abundance of modern food retail outlets in parallel with economic development. Although these trends were marked in some countries, other countries showed a relatively low number and low rate of growth of supermarkets, notably in south Asia. The present study was limited by having data on supermarkets only, without data on  other modern food outlets, such as convenience stores, which would also have been relevant to include. This was precluded because firstly, national‐level data from the Food Systems Dashboard excludes these and, secondly ‘supermarket’ is a more reliable search term in OpenStreetMap, there being no one definition/tag for ‘convenience’ retailing, making it harder and less reliable to extract data on convenience retailing in our study. This is a limitation of existing available datasets and something to be considered in future studies.

Other indicators of modernising food environments, such as trans‐national fast‐food chains, are also rapidly expanding in LMICs across the Asia‐Pacific region (Baker & Friel, 2016). This expansion bring further concerns about increasing accessibility of ultra‐processed foods (Baker & Friel, 2016; Sievert et al., 2019).

While modern retail outlets are increasing in availability and accessibility, consumer choices continue to play a key role in the uptake of modern or traditional food retailers, food purchasing behaviours and individual or household food consumption (Herforth & Ahmed, 2015). Affordability may be a barrier to supermarket use regardless of proximity. Households with low incomes may prefer to purchase from informal food vendors on a daily basis, rather than buy food from the nearest supermarket (Crush et al., 2019; Wertheim‐Heck & Raneri, 2019). Many consumers choose to frequent traditional or wet markets which can offer benefits in the freshness of products, affordability and accessibility for consumers of lower income (Suryadarma et al., 2010). Conversely, time poverty and city living may lead to greater consumption of pre‐prepared or processed foods. Among working women in urban slums of East Jakarta, purchase of ready‐made foods was preferred to cooking at home because of time poverty (Sufyan et al., 2019). Time‐constrained lifestyles and financial concerns were associated with less healthy food choices in Korean city dwellers (Kim et al., 2016).

In some cases, modern retail facilities can play a complementary role to more traditional markets when urban consumers adopt ‘cross‐platform’ shopping (Si et al., 2019). For households that are able to buy in bulk, supermarkets can offer financial savings compared to smaller retail outlets, but this also require space for food storage or refrigeration.

Evidence of associations between increasing density and availability of modern food retail outlets and increased consumption of processed foods among urban communities is mixed (Westbury et al., 2021). Some studies in Asia report negative associations between increased use of supermarkets and consumption of nutritious foods (Deakin University VicHealth & UNICEF, 2021; Farrell et al., 2021). A positive contribution of supermarkets is that they may result in higher consumption of a more diverse range of nutritious foods, as well as higher standards of hygiene and food safety (Deakin University VicHealth & UNICEF, 2021; FAO, UNICEF, et al., 2021). The potential risk of modern food retailers is the increased availability and accessibility of highly processed foods in the urban food environment at affordable prices (Baker et al., 2020; Deakin University VicHealth & UNICEF, 2021).

In sum, both modern and traditional retail food outlets have benefits but also potential risks in relation to nutritious and healthy diets. Modernisation of food retail outlets in LMICs could offer opportunities to guide retailers to provide affordable, accessible and nutritious options. If managed appropriately, modern and traditional or informal food outlets could provide complementary platforms for attaining healthy diets. The most important considerations for future actions are that urbanising food environments retain affordable and accessible nutritious foods to ensure the nutrition and food security of the population.

### 4.2 The concept of food deserts applied to the Asia‐Pacific region

The second aim of this paper was to examine how the concepts of food deserts and food swamps apply to urban food environments in the Asia‐Pacific and identify factors to mitigate the risks of creating food deserts and food swamps in future. Key differences of Asia‐Pacific cities are the presence of informal food vendors, the potential for self‐production or urban agriculture and the increased vulnerability of many cities to physical, environmental and socio‐economic shocks.

In the context of LMICs, the concept of ‘food deserts plus’ has been used to go beyond describing food deserts merely in terms of spatial dimensions such as access to a single food retail type (supermarkets). ‘Food deserts plus’ has been proposed to include a wider array of physical, social, and political dimensions of the food environment, to generate better insight, along with with a better understanding of the social‐cultural richness of the food environment across the region (Wagner et al., 2019). Similarly, ‘nutrition deserts’ may apply to LMICs in the Asia Pacific region. These are described as affecting the urban poor through the process of modernisation which displaces fresh food outlets with potentially negative consequences on health (Wertheim‐Heck & Raneri, 2019). Other important factors to consider include the spatial mobility of informal food vendors and poorer consumers; changing patterns of food security over time; and the differences between households with different exposure to food insecurity (Wagner et al., 2019). The distinctive features of Asia‐Pacific cities (informal food retailers, self‐production of food and building resilient food environments) are central to the future food security and nutrition of these urban centres.

### 4.3 Strengthening the resilience of urban food environments: Lessons learnt from the impact of COVID‐19 on informal vendors in three mega‐cities in the Asia‐Pacific

Many urban food environments in the Asia‐Pacific region are vulnerable to physical, environmental or socio‐economic shocks. Recommendations from the case study of the impact of COVID‐19 on informal food vendors further underline the importance of strengthening resilience through shorter food supply chains, using agri‐food innovation across towns and promoting food production in cities and surrounding areas, in line with global goals (FAO, 2019). Resilient food environments can also be built through supporting the income and livelihoods of producers, wholesalers and retailers and protecting access to healthy foods for poor urban populations. Suggested support mechanisms from the case study of urban informal food outlets and street food vendors during the COVID‐19 pandemic included strenghtening customer economic access and supporting informal sector vendors to diversify into online retailing, among other recommendations. City and local governments can be key enablers in mitigating the effects of economic, social and physical shocks in the food environment as demonstrated by responses to the COVID‐19 pandemic (FAO, 2020b).

## 5 CONCLUSIONS AND RECOMMENDATIONS

Urbanisation and economic development across the Asia‐Pacific region are leading to modernisation of food environments. Many cities in the region, especially those in LMICs, are undergoing rapid change. Both modern and traditional food outlets bring benefits and risks to food security and nutrition of low income populations. What is important is that cities provide healthy food environments, that is, environments that create the conditions that enable and encourage people to access and choose healthy diets (FAO, 2016). This is particularly important for nutrition of women and children who have greater vulnerability at times of economic or environmental shocks and pandemics (Kalbarczyk et al., 2022; Osendarp et al., 2021; Picchioni et al., 2021).

Important features of cities in the Asia‐Pacific region are the traditional and informal food outlets including wet markets and street food vendors and the potential for self‐production and urban agriculture. These features can be supported and promoted to allow them to coexist with modern retail outlets. Collectively, these outlets can create food environments providing affordable, accessible, varied and nutritious ranges of foods.

More research is needed on how the physical food environment influences the accessibility and affordability of nutritious foods for different socio‐economic groups in the urban population (Turner et al., 2020). Insights are needed on how consumers shop across the range of outlets including modern retailers, wet markets, informal vendors and street food outlets. Studies of street food vendors have focussed more on issues of hygiene and contamination and less on consumption patterns and preferences of customers (Abrahale et al., 2019). Better understanding is needed of purchasing practices of consumers either at the food outlet level (WHO, 2019) or household and individual level, and the contribution of different outlets to dietary intakes (Wertheim‐Heck & Raneri, 2019).

Similarly, research on the urban food environment and associated nutrition status or health outcomes for women and children is sparse for LMICs, and high quality evidence is needed (Turner et al., 2020). Few studies have systematically assessed the relationship between the urban food environment and specific dietary intakes such as fruit and vegetable consumption or ultra‐processed food consumption (Cheung et al., 2021; Kim et al., 2016; Leite et al., 2018; Sousa et al., 2021).

Cities represent wide‐ranging diversity and heterogenous populations with differing consumer behaviours. Caution then should be taken in overgeneralising with regards to an urban food environment and the relative importance of different components within and across different countries. The Asia‐Pacific region must also be considered in this regard with the wide diversity of economies, cultures, politics, geographies and trade links that define the food environment upon which urban dwellers depend.

## AUTHOR CONTRIBUTIONS

Emily Rousham developed the concept for the paper, critically analysed and synthesised literature, wrote the first draft of the manuscript with critical input from Sophie Goudet, Paula Griffiths, Jessica Blankenship, Martyn Clark, Michelle Latham, Swan Pyae Oo. Sophie Goudet developed the concept for the paper. Martyn Clark, Michelle Latham and Swan Pyae Oo extracted and analysed secondary data, critically analysed and synthesised literature. Sonja Read extracted data from the Rapid Market Assessment with supervision of Sophie Goudet. All authors read and approved the final version of the manuscript.

## CONFLICT OF INTEREST STATEMENT

The authors declare no conflicts of interest.

## Supporting information

Supporting information.

## Data Availability

The data that support the findings of this study were derived from the following resources available in the public domain: Food Systems Dashboard https://www.foodsystemsdashboard.org/; WorldPop Open Spatial Demographic Data and Research https://www.worldpop.org/, and OpenStreetMaps https://www.openstreetmap.org/. Data from the Emergency Market Mapping and Analysis report are available from Dikoda https://dikoda.com/ on reasonable request. Secondary data taken from the EMMA report was collected underthe Global Code of Conduct for Research in Resource‐Poor Settings. The remaining data used are from open access GIS tools andsecondary data sources.
