# Supplementary material for: Resilience and vulnerabilities of urban food environments in the Asia‐Pacific region
Source: Matern Child Nutr. 2023 Apr 25;22(1):e13513. doi: 10.1111/mcn.13513 (PMC12647982; doi:10.1111/mcn.13513)
Supplement: Supplementary file 1 — Supporting information. [file MCN-22-e13513-s001.docx]

**Supplementary materials: *Resilience and vulnerabilities of urban food environments in the Asia-Pacific region*.**

Emily K. Rousham, Martyn Clark^,^, Michelle Latham, Swan Pyae Oo, Sonja Read, Paula Griffiths, Jessica Blankenship, Sophie Goudet

**Supplementary Table 1.** Summary of data on availability and accessibility of supermarkets in major cities of 23 countries in the Asia-Pacific region showing the proportion of the city population within a 5 minute and 20 minute walking distance to a supermarket†

| City | Country | Total supermarkets in 50 km city buffer (n) | Total population in 50 km city buffer (n) | Supermarkets per 100,000 population | Population living within 5 min walking distance to supermarket (n) | Population within 5 min walking distance to supermarket out of total city population (50 km buffer) (%) | Population living within 20 min walking distance to supermarket (n) | Population within 20 min walking distance to supermarket out of total city population (50 km buffer) (%) |
| --- | --- | --- | --- | --- | --- | --- | --- | --- |
| Seoul | Republic of Korea | 2,313 | 24,338,028 | 9.5 | 8,223,477 | 33.8 | 22,458,951 | 92.3 |
| Beijing | China | 404 | 19,877,686 | 2 | 1,911,150 | 9.6 | 10,807,318 | 54.4 |
| Thimphu | Bhutan | 24 | 269,906 | 8.9 | 10,984 | 4.1 | 13,057 | 4.8 |
| Colombo | Sri Lanka | 434 | 7,472,821 | 5.8 | 1,293,142 | 17.3 | 4,475,407 | 59.9 |
| Kathmandu | Nepal | 170 | 4,675,225 | 3.6 | 846,734 | 18.1 | 2,635,105 | 56.4 |
| Islamabad | Pakistan | 113 | 8,691,707 | 1.3 | 362,202 | 4.2 | 2,634,639 | 30.3 |
| Kabul | Afghanistan | 51 | 6,438,954 | 0.8 | 469,476 | 7.3 | 3,390,571 | 52.7 |
| Dhaka | Bangladesh | 205 | 29,440,618 | 0.7 | 3,407,667 | 11.6 | 12,816,591 | 43.5 |
| Tehran | Iran | 954 | 13,776,608 | 6.9 | 1,027,258 | 7.5 | 4,973,267 | 36.1 |
| New Delhi | India | 159 | 32,693,158 | 0.5 | 1,637,534 | 5 | 12,645,102 | 38.7 |
| Kuala Lumpur | Malaysia | 964 | 7,532,795 | 12.8 | 2,262,767 | 30 | 6,964,645 | 92.5 |
| Vientiane | Lao PDR‡ | 124 | 1,608,487 | 7.7 | 134,981 | 8.4 | 318,713 | 19.8 |
| Naypyidaw | Myanmar | 35 | 924,608 | 3.8 | 23,286 | 2.5 | 256,209 | 27.7 |
| Hanoi | Viet Nam | 505 | 14,731,018 | 3.4 | 2,147,436 | 14.6 | 4,852,712 | 32.9 |
| Manila | Philippines | 949 | 28,026,170 | 3.4 | 5,679,377 | 20.3 | 23,425,469 | 83.6 |
| Phnom Penh | Cambodia | 170 | 5,725,385 | 3 | 743,441 | 13 | 1,786,674 | 31.2 |
| Bangkok | Thailand | 316 | 11,269,970 | 2.8 | 900,721 | 8 | 5,805,186 | 51.5 |
| Jakarta | Indonesia | 285 | 33,103,406 | 0.9 | 1,672,218 | 5.1 | 15,623,473 | 47.2 |
| Apia | Samoa* | 23 | 35,000 | 65.7 | 19,202 | 54.9 | 30,040 | 85.8 |
| Palikir | Micronesia* | 6 | 21,670 | 27.7 | 6324 | 29.2 | 7,195 | 33.2 |
| Port Vila | Vanuatu* | 23 | 89,968 | 25.6 | 28,465 | 31.6 | 37,926 | 42.2 |
| Honiara | Solomon Islands* | 19 | 116,955 | 16.2 | 21,849 | 18.7 | 39,418 | 33.7 |
| Port Moresby | Papua New Guinea* | 53 | 364,117 | 14.6 | 68,873 | 18.9 | 79,495 | 21.8 |

†Data sources: City population data were extracted from www.worldpop.org (2020) and supermarket data were extracted from OpenStreetMap (<https://www.openstreetmap.org).‡> PDR, People’s Democratic Republic.*A 25 km buffer for the city population and number of supermarkets was applied in the case of the Pacific Island States.

**Supplementary Table 2:** Recommendations for interventions to increase resiliance of informal food retail outlets in urban slums within the three cities of Jakarta in Indonesia, Dhaka in Bangladesh and Quezon City in the Philippines based on Emergency Market Mapping Assessment during COVID-19

|  |  | **Relevance to poor urban areas of the three cities included in the survey** | | |
| --- | --- | --- | --- | --- |
| **Intervention objective** | **Intervention recommendations** | **Jakarta** | **Dhaka** | **Quezon City** |
| Improve purchasing power of consumers, thereby supporting the economy and supply chains | - Cash and voucher assistance and livelihoods support targeting poor urban areas, especially those who have lost their jobs | 🗸 | 🗸 | 🗸 |
| Support small-to-medium enterprises and street vendors’ sustainability and prevent them from closing down | - Offer financial support such as business loans or grants with low or no interest; tax reductions or exemptions - Advertise existing support schemes more widely, especially those that are accessible to unregistered vendors - Financial support was the most desired assistance sought by informal vendors | 🗸 | 🗸 |  |
| Increase consumer and local business confidence in safe and hygienic preparation of street foods during COVID-19 outbreaks | - Promote hygienic preparation and sales of street foods (e.g. appropriate packaging) and appropriate use of protective equipment - Skills training with financial support to adapt to external shocks. Regulation and safety inspections including provision of protective equipment, protocols on social distancing, cold chain equipment, WASH facilities and support for health inspections and certification | 🗸 | 🗸 |  |
| Facilitate consumer access to nutritious foods in poor urban areas and support livelihoods of street food vendors | - Link street food vendors with existing government interventions to provide low-cost foods in poor areas | 🗸 |  | 🗸 |
| Capacity building for street food vendors to build resilience | - Training for street food vendors to diversify or switch to selling other products - Support food vendors to use technology to move to online orders via collective channels (sales platform at low cost) or improve connections and communication with consumers - Support food businesses to design alternative methods for customer access during curfews or restrictions on movement | 🗸 |  | 🗸 |
| Stabilise food prices | - Government and municipalities to support price caps for key commodities during crisis | 🗸 |  |  |
| Interventions to strengthen market linkages for small retailers and street food vendors | - Facilitate market linkages between producers and retailers especially for smaller retailers and street food vendors to ensure that supply chains are maintained - Facilitate movement of goods across areas within cities - Enhance linkages between different market actors through technology to create COVID-safe connections |  | 🗸 | 🗸 |
| Continue current financing mechanisms for street food vendors and expand inclusion criteria | - Extend registration of informal street food vendors (e.g. for those not currently registered or those not resident in Quezon City) under the Quezon City Market Development and Administration Department (MDAD), to increase the number of vendors receiving financial assistance and capacity building |  |  | 🗸 |
| Minimize disruption to supply chains | - Ensure good transport systems for fresh, raw and perishable foods and cold storage |  | 🗸 |  |
| Minimize food losses | - Improve storage facilities and cold supply chains to factor for disruptions and decrease loss of perishable foods |  | 🗸 |  |
| Cross-cutting | - Consider access of unregistered vendors and actors to any intervention; where registration is needed, explore alternative options such as certificates from local neighbourhood leaders, support for vendors to register, or grouping of informal actors for targeting as a cluster |  | 🗸 |  |
